# Lipopolysaccharide potentiates hyperthermia-induced seizures

**DOI:** 10.1002/brb3.348

**Published:** 2015-06-02

**Authors:** Baik-Lin Eun, Jayne Abraham, Lauren Mlsna, Min Jung Kim, Sookyong Koh

**Affiliations:** 1 Department of Pediatrics, Korea University College of MedicineSeoul, Korea; 2 Department of Pediatrics, Ann and Robert H. Lurie Children’s Hospital of Chicago Stanley Manne Children’s Research Institute, Northwestern University Feinberg School of MedicineChicago, Illinois

**Keywords:** Brain inflammation, cytokines, febrile seizures, lipopolysaccharide, microglia, temporal lobe epilepsy

## Abstract

**Background:**

Prolonged febrile seizures (FS) have both acute and long-lasting effects on the developing brain. Because FS are often associated with peripheral infection, we aimed to develop a preclinical model of FS that simulates fever and immune activation in order to facilitate the implementation of targeted therapy after prolonged FS in young children.

**Methods:**

The innate immune activator lipopolysaccharide (LPS) was administered to postnatal day 14 rat (200 *μ*g/kg) and mouse (100 *μ*g/kg) pups 2–2.5 h prior to hyperthermic seizures (HT) induced by hair dryer or heat lamp. To determine whether simulation of infection enhances neuronal excitability, latency to seizure onset, threshold temperature and total number of seizures were quantified. Behavioral seizures were correlated with electroencephalographic changes in rat pups. Seizure-induced proinflammatory cytokine production was assessed in blood samples at various time points after HT. Seizure-induced microglia activation in the hippocampus was quantified using Cx3cr1^GFP/+^ mice.

**Results:**

Lipopolysaccharide priming increased susceptibility of rats and mice to hyperthemic seizures and enhanced seizure-induced proinflammatory cytokine production and microglial activation.

**Conclusions:**

Peripheral inflammation appears to work synergistically with hyperthermia to potentiate seizures and to exacerbate seizure-induced immune responses. By simulating fever, a regulated increase in body temperature from an immune challenge, we developed a more clinically relevant animal model of prolonged FS.

## Introduction

Fever is the most frequent cause of seizures in children worldwide (Shinnar and Glauser 2002; Wang et al. 2010). It is estimated that 5,000 to 10,000 cases of febrile status epilepticus occur annually in the United States, accounting for 25–30% of all childhood status epilepticus and over 70% of status in the second year of life (Hauser and Kurland 1975; Nelson and Ellenberg 1976; Shinnar et al. 2001). Not only are prolonged febrile seizures (FS) associated with hippocampal injury (VanLandingham et al. 1998) and sclerosis – severe hippocampal neuron loss and gliosis (Dube et al. 2010; McClelland et al. 2011) – they may also increase the risk for the development of mesial temporal lobe epilepsy later in life. A prospective, longitudinal, multicenter study of the consequences of prolonged FS (FEBSTAT) has identified promising short-term surrogate biomarkers to identify the children at the highest risk of developing future hippocampal sclerosis and mesial temporal lobe epilepsy (Lewis et al. 2014), opening the possibility for novel treatment strategies aimed at preventing the short- and long-term consequences of febrile seizures. Preclinical efficacy evaluation of potentially anti-epileptogenic therapy for patients at risk is urgently needed and requires a clinically relevant animal model of prolonged febrile seizures.

Presently, the established experimental model of FS consists of hyperthermia-induced seizures in Postnatal day (P) 10 rat pups (Baram et al. 1997). While this model of FS is appropriately age-specific (Dube et al. 2000) and leads to increased risk for development of spontaneous seizures in adulthood (Dube et al. 2010), seizures are triggered by raising core body temperature (T_b_) using an external heat source rather than by endogenous fever. This distinction is essential since different physiologic mechanisms underlie fever and hyperthermia (Berg 1993). Fever is a regulated increase in body temperature that results from an immune challenge. It involves peripheral and central nervous system cytokine signaling and generation of prostaglandins throughout the brain that activate heat conservation and production pathways (Oladehin and Blatteis 1997; van Dam et al. 1998; Saper 1998). In contrast, hyperthermia is an increase in body temperature that results from an excessive heat load that outstrips the organism’s ability to cool itself and involves mobilization of heat dissipation mechanisms in an attempt to reduce body temperature (Berg 1993; Ostberg et al. 2000). Because FS are intrinsically associated with inflammatory processes – often related to ear infection or systemic HHV6 infection in infants and young children (Hall et al. 1994) – we aim to develop an animal model of FS that simulates fever and inflammation as opposed to hyperthermia alone. By combining lipopolysaccharide (LPS), a Toll-like Receptor 4 (TLR4) ligand and activator of the innate immune response, with hyperthermia-induced seizures in P14 rat and mouse pups, we demonstrate that simulation of peripheral infection by injection of LPS prior to hyperthermia enhances neuronal excitability, seizure-induced proinflammatory cytokine production, and microglial activation.

## Methods

### Animals

In *Experiment 1 and 2,* P14 Long Evans rats (Charles River Laboratories, Wilmington, MA) were used to assess seizure susceptibility and severity, electrographic (EEG) correlates of seizures as well as peripheral cytokine production.

In *Experiment 3,* P14 C57Bl/6 mice (Jackson Laboratories, Farmington, CT) were used to assess seizure susceptibility, severity, and peripheral production of cytokines. Cx3cr1^GFP/+^ transgenic mice (obtained as a gift from Dr. Jaime Grutzendler, New Haven, CT) were used to assess microglia activation. In Cx3cr1^GFP/+^ mice, the fractalkine chemokine receptor has been replaced by a green fluorescent protein (GFP) reporter gene by targeted deletion via homologous recombination in embryonic stem cells (Jung et al. 2000; Davalos et al. 2005). All animals were group housed in polypropylene cages and maintained at 21°C with ad libitum access to water and rodent chow. All procedures were in accordance with the National Institutes of Health Guidelines for the Care and Use of Laboratory Animals and were approved by the Stanley Manne Children’s Research Institute Institutional Animal Care and Use Committee.

### Experimental design

#### Experiment 1

**Figure 1:**
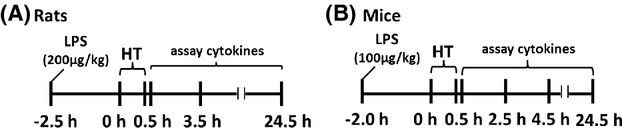
Experimental protocol for induction of infection-associated febrile seizures. (A) Rats were injected with lipopolysaccharide (LPS) (200 μg/kg, *i.p*.) 2.5 h prior to induction of hyperthermic seizures, using a hair dryer (Experiment 1) or heat lamp (Experiment 2). Blood cytokine levels were assayed at 0, 3, and 24 h after hyperthermic seizures. (B) Mice were injected with lipopolysaccharide (LPS) (100 *μ*g/kg, *i.p*.) 2 h prior to induction of hyperthermic seizures using a heat lamp (Experiment 3). Blood cytokine levels were assayed at 0, 2, 4, and 24 h after hyperthermic seizures.

To allow direct comparisons between a well-established experimental FS model and our model of infection-associated FS, hyperthermic seizures (HT) were induced using a hairdryer according to the protocol developed in laboratory of Dr. Tallie Baram (Dube et al. 2005). A total of 68 P14 male Long Evans rat pups were used. Animals were randomly divided into four groups: (1) LPS-normothermia (LPS only), (2) LPS-hyperthemia (LPS-HT), (3) PBS-hyperthemia (HT only), and (4) normothermia controls. LPS only and LPS-HT animals were primed with LPS (Escherichia coli*,* serotype 0127:B8; Sigma Chemicals Co., St. Louis, MO; 200 *μ*g/kg, i.p.) 2.5 h prior to induction of hyperthermia to simulate fever (Fig.1A). An additional group of rat pups (*n *=* *3 HT; 3 LPS + HT) were implanted with EEG head mounts on P10 and monitored during seizure induction via a tethered data acquisition system on P14 in order to confirm the electrographic correlates of behavioral seizures.

#### Experiment 2

In order to maintain greater control of temperature, minimize dehydration, tissue trauma, and hyperventilation (Schuchmann et al. 2008) and in preparation for a subsequent transition to a murine model of infection-associated FS, we adapted the model by using a heat lamp to induce hyperthermia in rat pups, as used by Oakley et al. in mice (Oakley et al. 2009). As our intention was to demonstrate replication of behavioral and electrographic seizure semiology in rats using this method of hyperthermia induction, all animals were primed with LPS (200 *μ*g/kg, i.p.) 2.5 h prior to induction of hyperthermic seizures (Fig.1A). Rat pups (*n *=* *4) were implanted with EEG head mounts on P11 and monitored during seizure induction on P14.

#### Experiment 3

In order to capitalize on transgenic and knockout mouse technology, we extended our infection-associated FS model to mice. A total of 85 P14 C57BL/6 mice and 23 Cx3cr1^GFP/+^ mice were used. Pups were divided into four groups (LPS only, LPS-HT, HT-only, and normothermia control). LPS groups were primed with LPS (100 *μ*g/kg, i.p.) 2 h prior to induction of hyperthermia, using the heat lamp protocol (Fig.1B). Twenty-four hours after hyperthermia induction, Cx3cr1^GFP/+^ mice were euthanized and their brains collected for quantification of microglial activation.

### Lipopolysaccharide treatment

#### Experiments 1 and 2

To determine the dose of LPS and optimal time interval between LPS injection and hyperthermic seizure induction, rat pups were separated from their dams, placed in a 30°C incubator, and injected with 25, 50, 100 or 200 *μ*g/kg LPS (50 *μ*g/mL dissolved in pyrogen-free saline, *n *=* *2–6/group). T_b_ was recorded at 15 min intervals for 3 h (data not shown). After 2.5 h, *T*_b_ was noted to be elevated relative to controls in rats injected with 200 *μ*g/kg LPS, but was not elevated in groups that received lower dosages.

#### Experiment 3

A dose of 100 *μ*g/kg LPS administered 2 h prior to hyperthermic seizure induction was chosen for mice based on published studies demonstrating elevated temperature (Lawrence et al. 2012) and levels of cytokine expression (Skelly et al. 2013) at that dosage.

### Seizure induction

#### Experiment 1

Hyperthermic seizures (HT) were induced according to the protocol as described previously (Dube et al. 2005). P14 rat pups were held in an incubator set to 30°C for at least 15 min prior to seizure induction. Pups were kept at 30°C instead of at room temperature (25°C) before seizure induction because 30°C is normal nest temperature and within the thermal neutral zone for pups at this age (Heida et al. 2004). LPS-only and normothermia controls remained in the incubator for the duration of the experiment. To induce seizures, pups were placed in a 3-L glass chamber with a cloth-covered floor, and a regulated stream of moderately heated air was directed over the top of the container using a commercial hair dryer. T_b_ was measured every 2 min using a rectal probe (RET-4; Physitemp, Clifton, NJ) connected to a digital thermocouple thermometer (WD-35427-20, Oakton Instruments, Vernon Hills, IL). T_b_ was elevated by 1°C/min until a seizure occurred or temperature exceeded 41.5°C, at which point the animal was removed from the chamber and placed on a cool metal surface for at least 2 min and until T_b_ was below 41°C. Behavioral seizures consisted of sudden movement arrest followed by facial automatisms (chewing), limb clonus, clonic jerks, limb stiffening, and finally generalized tonic-clonic (GTC) seizures and body flexion. Hyperthermia (≥39.0°C) was maintained for 30 min, with a total seizure duration of at least 20 min. Following hyperthermia, animals were submerged in room temperature water, hydrated orally, and placed on a cool metal surface until T_b_ returned to normal for age range (32–34°C). All animals were returned to their dams, with total separation time kept under 4 h.

#### Experiment 2

Seizures were induced in a Plexiglas chamber (13 × 24 × 12.6 cm) heated by a heat lamp positioned approximately 10 cm above the chamber. T_b_ was elevated by 0.5°C every 2 min until a seizure occurred or temperature exceeded 41.5°C. Following 30 min of hyperthermia, animals were cooled on a metal surface, rehydrated by saline injection, and returned to their dams.

#### Experiment 3

Hyperthermic seizures were induced in mouse pups on P14 as described in Experiment 2. Behavioral seizures consisted of tail shaking, limb clonus, falling, shaking, and finally GTC seizures. Following 30 min of hyperthermia, mice were cooled on a metal surface, rehydrated by saline injection, and returned to their dams. In doing so, death during and immediately after hyperthermia was minimized, and mortality maintained below 10% (3/52; 6% mortality rate).

### EEG recording

#### Experiments 1 and 2

On P10-11, rat pups were implanted with *mouse* EEG headmounts (#8201, Pinnacle Technology, Lawrence, KS) as described previously (Radzicki et al. 2013). Briefly, rats were aligned in a stereotaxic apparatus and anesthetized with isoflurane/O_2_. A skin incision was made and the headmount placed on the exposed surface of the skull. Two pairs of screw electrodes (#8209, Pinnacle Technology, Lawrence, KS) were drilled through the skull to rest on the cerebral cortex, positioned bilaterally anterior to bregma and bilaterally anterior to the lambdoid suture. A two-part epoxy (SEC1233, Resinlab, Germantown, WI) was used to ensure electrical conductivity between the screw electrodes and headmount. Dental acrylic was applied over the screws and headmount to secure and insulate the apparatus. The skin incision was sutured closed and the pup was placed on a heating pad until consciousness returned, at which point it was returned to its dam. Animals were allowed to recover for at least 3 days prior to seizure induction and data acquisition. EEG was recorded using the Pinnacle 4100 recording system (Pinnacle Technology, Lawrence, KS). EEG analysis was performed by an electroencephalographer (SK) who was blinded to the identification of animals.

### Peripheral cytokine production

At different time points after hyperthermia, a subset of rats (Experiment 1; *n *=* *3–7/group; *t* = 0, 3, 24 h) and mice (Experiment 3; *n *=* *3–14 per group; *t* = 0, 2, 4, 24 h) were deeply anesthetized with pentobarbital (100 mg/kg, *i.p*.) and had blood collected by transcardiac puncture. Total protein concentration was calculated using the BCA assay kit (Pierce, Rockford, IL). Levels of 7 cytokines, IL-1*β*, IL-12p70, IFN-*γ*, IL-6, KC/GRO, IL-10, and TNF-*α*, were measured using ELISA-based commercially available kits (Meso-Scale Discovery, Gaithersburg, MD). Samples were analyzed in duplicates and compared with controls. Plates were analyzed using the SECTOR Imager 2400 (Meso-Scale Discovery, Gaithersburg, MD).

### Microglial activation

Cx3cr1^GFP/+^ mice were deeply anesthetized and perfused transcardially with PBS followed by ice-cold 4% paraformaldehyde/0.1 mol/L sodium phosphate buffer. Brains were harvested, postfixed with 4% paraformaldehyde/30% sucrose solution overnight, and mounted on a freezing microtome. Thereafter, 40 *μ*m horizontal sections were cut, and every 6th section collected and mounted on slides for microscopic examination. At least six hippocampal sections per brain from at least four animals per group were selected for quantification. The anterior commissure was used as a specific landmark to match sections across experiments. Images were captured digitally at 20× magnification, converted to gray scale, and areas of positively labeled green fluorescent cells were highlighted within the hilus of the hippocampus to allow consistent comparison between controls and KA animals over time. Quantification threshold was held constant for all specimens within each experimental group and quantified using ImageJ (1.43 *μ*, Public Domain, NIH) by a single observer (J.A.) who was blinded to the treatment groups.

### Statistical analysis

Student’s *t*-tests (Prism v. 5.0, GraphPad) were used to compare temperature, latency to seizure onset, and seizure threshold temperature between groups. A two-way analysis of variance (ANOVA) with post hoc Bonferroni-corrected t-tests was used to compare cytokine levels between all groups at various time points. A one-way ANOVA with post hoc Tukey-corrected t-tests was used to compare differences in microglia activation among experimental groups. For each statistical test, a parametric test was chosen. Data are expressed as the mean ± standard error of the mean (SEM) and significance was defined as *P *<* *0.05 for all tests.

## Results

### Experiment 1. Hairdryer induction protocol in rats: LPS increases susceptibility to hyperthermia-induced seizures and exacerbates seizure-induced cytokine production

**Figure 2:**
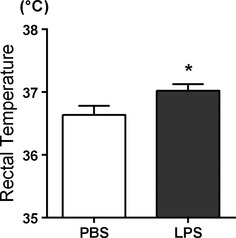
Baseline body temperatures of P14 rats are elevated 2.5 h after intraperitoneal injection of 200 *μ*g/kg lipopolysaccharide (LPS) (PBS: 36.6 ± 0.1°C, *n *=* *22; LPS: 37.0 ± 0.1°C, *n *=* *23; Student’s *t*-test, *P <* *0.05).

**Figure 3:**
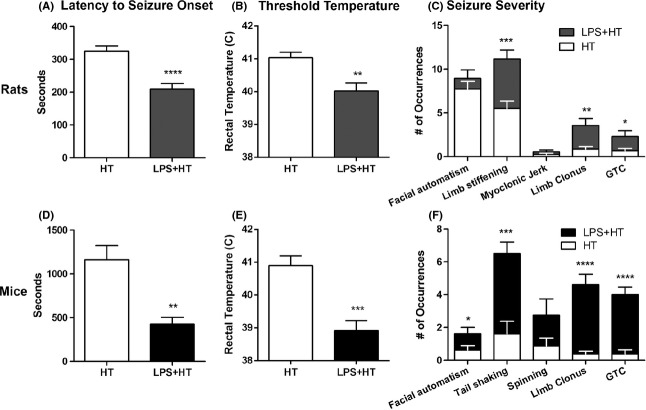
Priming with lipopolysaccharide (LPS) increases the susceptibility of P14 rat and mouse pups to hyperthermic seizures. (A, D) Latency to seizure onset is significantly decreased in LPS + HT pups compared to pups that experienced hyperthermic seizures (HT) alone, for both rats (HT: 325.0 ± 16.1, *n *=* *22; LPS + HT: 209.0 ± 17.5, *n *=* *23; *P *<* *0.0001) and mice (HT: 1162 ± 160.8, *n *=* *3; LPS + HT: 425.0 ± 79.8, *n *=* *7; *P *<* *0.01) (B, E) Seizure threshold temperature is significantly lower in LPS + HT pups compared to pups that experience HT alone, in both rats (HT: 41.0 ± 0.2°C, *n *=* *22; LPS + HT: 40.0 ± 0.2°C, *n *=* *23; *P *<* *0.01) and mice (HT: 40.9 ± 0.3°C, *n *=* *6; LPS + HT: 38.9 ± 0.3°C, *n *=* *8; *P *<* *0.001). (C, F) LPS + HT pups exhibited a greater frequency of seizure behaviors during the period of hyperthermia compared to pups experiencing HT alone (*P <* *0.05, **P <* *0.01, ***P <* *0.001 & ****P <* *0.0001). Student’s t-tests were used to compare groups.

**Figure 4:**
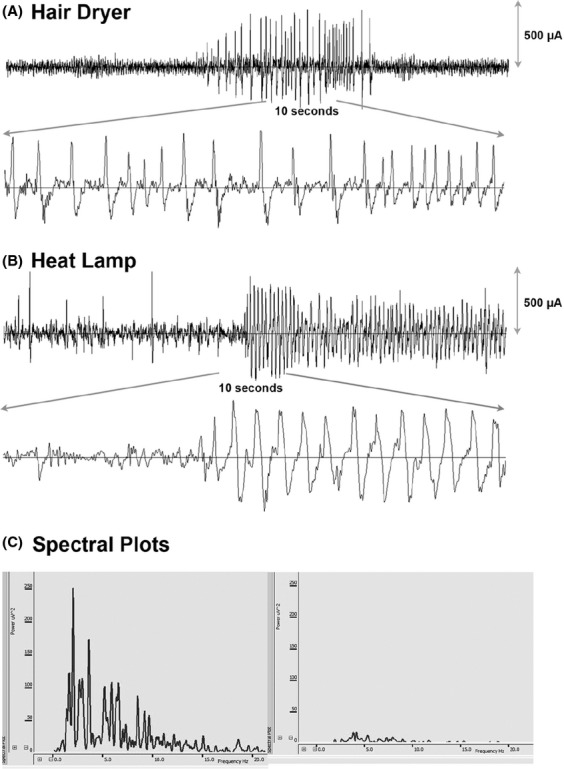
LPS-primed hyperthermic seizures induced using either the hair dryer (A) or heat lamp (B) protocol have identifiable electroencephalographic correlates. Generalized tonic clonic seizures (GTCs) consistently manifested as 60-95 s-long runs of high voltage rhythmic ictal discharges in P14 rat pups. (C) Spectral analysis (Sirenia Seizure Pro v. 1.6.6, Pinnacle Technology) from 10 s epochs during (left panel) and after (right panel) a seizure induced by heat lamp shows a marked increase in low frequency activities during a seizure compared to the postictal state. LPS, lipopolysaccharide.

**Figure 5:**
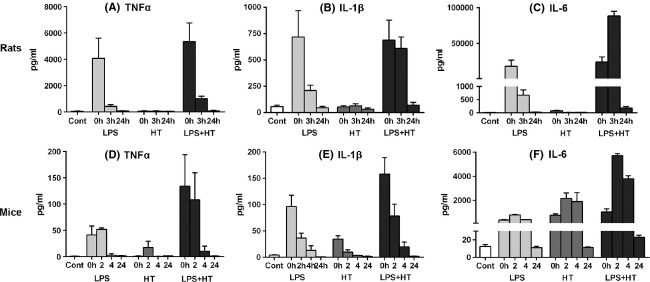
Priming with lipopolysaccharide (LPS) prior to hyperthermic seizures enhances seizure-induced production of TNF-*α*, IL-1*β*, and IL-6 in the blood. In rats, there was a significant difference in all three proinflammatory cytokines levels between treatment groups at different time points (TNF-*α*: *P *<* *0.01; IL-1*β*: *P *<* *0.001; IL-6: *P *<* *0.0001, two-way ANOVA). 0 h = 3 h post-LPS injection and 0.5 h postseizure induction. (A) Compared to controls, a significant elevation of TNF-*α* was only detected at 0 h in the LPS + HT group (*P *<* *0.01). TNF-*α* was significantly higher in the LPS + HT group compared to the HT-only group at 0 h (*P *<* *0.0001). (B) IL-1*β* was modestly elevated in the LPS-only and LPS + HT groups. IL-1*β* was significantly higher in the LPS + HT group compared to the HT-only group at 0 h (*P *<* *0.01) and 3 h (*P *<* *0.05). (C) A significant increase in IL-6 was noted only in the LPS + HT group at 3 h (*P *<* *0.0001). IL-6 was significantly higher in the LPS + HT group compared to the HT-only group at 0 h (*P *<* *0.01) and 3 h (*P *<* *0.0001). In mice, there was a significant difference in IL-1*β* (*P *<* *0.001, two-way ANOVA) and IL-6 (*P *<* *0.0001) between treatment groups at different time points. 0 h = 2.5 h post-LPS injection and 0.5 h post-seizure induction. (D) Minimal elevation of TNF-*α* was noted in the HT-only group, with only a modest elevation present in the LPS + HT group. (E) Compared to controls, there was a significant increase in IL-1*β* in both the LPS-only (*P *<* *0.01) and LPS + HT (*P *<* *0.0001) groups at 0 h. IL-1*β* was significantly higher in the LPS + HT group compared to the HT-only group at 0 h (*P *<* *0.01). (F) There was a significant increase in IL-6 in both the HT-only and LPS + HT groups at 2 h and 4 h (*P *<* *0.0001, all comparisons). IL-6 was significantly higher in the LPS + HT group compared to the HT-only group at 2 h (*P *<* *0.0001) and 4 h (*P *<* *0.01). Student’s t-tests were used to compare two groups. LPS, lipopolysaccharide; HT, hyperthermic seizures.

All rats from both HT (*n *=* *22) and LPS + HT (*n *=* *23) groups developed seizures that were confirmed by EEG in a select group of animals. Baseline temperatures were higher in LPS-injected rats than saline-treated rats 2.5 h after LPS injection and prior to experimental seizure (*P *<* *0.01) (Fig.2). Priming with LPS significantly decreased latency to seizure onset (*P *<* *0.0001) (Fig.3A) and seizure threshold temperature (*P *<* *0.01) (Fig.3B). Seizure semiology progressed from behavioral arrest, facial automatisms (chewing), limb stiffening, and myoclonic jerks to forelimb and hindlimb clonus and finally sudden loss of posture, flexion of body, and generalized convulsion (generalized tonic clonic seizures, GTCs). GTCs had consistent electrographic correlates manifested as 60–95 s-long runs of high-voltage rhythmic ictal discharges on EEG (Fig.4A). Throughout the period of hyperthermia, frequent interictal discharges of high-voltage spikes and slow wave or sharp wave discharges occurred either in runs or in isolation. LPS + HT rats exhibited a greater frequency of seizure behaviors during the period of hyperthermia compared to HT-only rats with significant increases in limb stiffening, limb clonus, and GTCs (Fig.3C). While all animals in the HT group survived (0% mortality), LPS-treated animals, including LPS-only and LPS-HT, had higher mortality (4.8% and 11.5%, respectively). These data demonstrate that peripherally administered LPS potentiates susceptibility to and severity of hyperthermia-induced seizures in P14 rats. Concomitant with increased seizure susceptibility, priming with LPS significantly activated seizure-induced production of proinflammatory cytokines IL-1*β*, IL-6, and TNF-*α* in the blood compared to HT-only (Fig.5A). Among the seven cytokines tested, these three cytokines were significantly elevated and consistently detectable in the blood samples. While HT alone failed to induce proinflammatory cytokines above baseline and LPS alone led to a transient increase in blood cytokine level 3 h after injection, LPS priming caused sustained cytokine production beyond 3 h after seizure induction in LPS + HT animals.

### Experiment 2. Heat lamp induction protocol in rats: Heat lamp-induced hyperthermic seizures manifest similarly to hairdryer-induced seizures

P14 rat pups responded to heat lamp-induced hyperthermia similarly to hyperthermia produced by the hair dryer protocol. Seizure behaviors progressed from behavioral arrest, facial automatisms (chewing), limb stiffening, myoclonic jerks to forelimb, hind limb clonus, and finally sudden loss of posture and GTCs. Electrographic correlates of GTC seizure were similar to those induced by hair dryer hyperthermia (Fig.4B).

### Experiment 3. Heat lamp induction protocol in mice: LPS priming worsens seizures and exacerbates cytokine production and microglia activation

**Figure 6:**
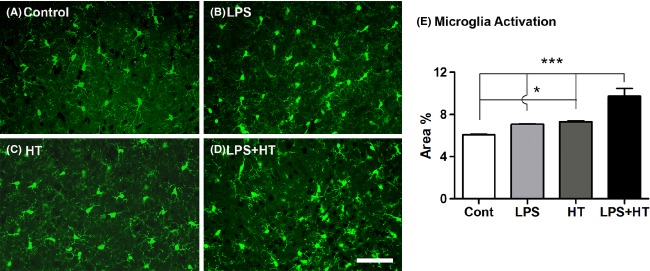
Lipopolysaccharide (LPS) potentiates seizure-induced microglial activation in Cx3cr1^GFP^^/+^ mice. (A–D) Representative hippocampal sections from normothermic control, LPS-only, HT-only, and LPS + HT mice at 24 h. Scale bar = 50 *μ*m. (E) Percent area of fluorescence for control (*n *=* *6), LPS-only (*n *=* *6), HT-only (*n *=* *6), and LPS + HT (*n *=* *5) groups. LPS + HT was significantly increased compared to control, LPS-only and HT-only groups (*P *<* *0.0001, one-way ANOVA with Tukey post hoc comparison, *P <* *0.05, ***P <* *0.001). LPS, lipopolysaccharide; HT, hyperthermic seizures.

Similar to the response seen in rats, LPS priming decreased seizure latency nearly twofold compared to hyperthermia alone (*P *<* *0.01) (Fig.3D). Seizure threshold temperature was reduced nearly 2°C in LPS-primed animals (*P *<* *0.001) (Fig.3E). LPS + HT mice sustained more severe seizures than HT-only mice. Seizure behaviors progressed from behavioral arrest, facial automatisms (chewing), tail shaking and/or spinning, to limb clonus,and finally sudden loss of posture and GTCs. LPS + HT mice exhibited significant increases in facial automatisms, tail shaking, limb clonus, and GTCs compared to mice that underwent HT alone. Concomitant with increased seizure susceptibility, LPS + HT significantly activated IL-1*β*, IL-6, TNF-*α* production in the blood, while LPS-alone or HT-alone had only modest and transient effects (Fig.5B). In addition, priming with LPS led to marked microglia activation 24 h following HT seizures in Cx3cr1^GFP/+^ mice (Fig.6). A significant increase in the area of fluorescent cells was noted in LPS + HT mice compared to control, LPS-only, and HT-only littermates (*P < *0.0001). Neither LPS alone nor hyperthermia alone led to changes in microglia activation.

## Discussion

In the present study, we demonstrated that (1) induction of a period of hyperthermia in P14 rat or mouse pups using either a hairdryer or heat lamp produces stereotyped seizures with consistent EEG correlates, (2) priming with the bacterial endotoxin LPS increases susceptibility of rat and mouse pups to hyperthermic seizures, and (3) LPS priming prior to hyperthermic seizure induction increases proinflammatory cytokine production and microglial activation. Together, these findings suggest that LPS potentiates hyperthermic seizures in rat and mouse pups and that the LPS + HT model recapitulates essential aspects of infection-associated FS that more closely mimic the clinical situation.

Lipopolysaccharide, a cell wall component of gram-negative bacteria, has been used to induce fever and simulate infection in numerous species. LPS activates toll-like receptor 4 (TLR-4), which is expressed on cells of the innate immune system. Subsequent downstream signaling via the MyD88 pathway leads to activation of NF-kB and release of proinflammatory cytokines. This in turn leads to induction of the enzyme cyclooxygenase-2 (COX-2) and increased production of prostaglandin E2, which causes fever (Lu et al. 2008). LPS has previously been shown to increase susceptibility to seizures induced by various proconvulsants, including kainic acid, pentylenetretrazole, and lithium-pilocarpine (Sayyah et al. 2003; Heida et al. 2004; Galic et al. 2008). In the present study, priming with LPS (200 *μ*g/kg) led to an increase in body temperature in rat pups and a decrease in seizure susceptibility and seizure threshold temperature. Our results differ from those of Auvin et al. (Auvin et al. 2007, 2009), in which priming with LPS did not increase T_b_ or hyperthermic seizure susceptibility in rat pups. This difference may be explained by the lower dose of LPS (10, 50, or 100 *μ*g/kg; 5 or 50 *μ*g/kg, respectively) used in these studies or could be due to differences in body temperature (30°C vs. room temperature) during LPS priming (Dupuis and Auvin 2015).

An experimental model of FS should capture the immune component of the clinical entity based on accumulating evidence for the involvement of inflammation and immune activation in FS and epilepsy. Children with febrile seizures, for example, are more likely to possess gene polymorphisms that lead to increased production of the proinflammatory cytokines IL-1*β* or IL-6 (Kanemoto et al. 2000; Virta et al. 2002; Nur et al. 2012). They also exhibit increased blood levels of IL-1*β*, IL-6 and the inflammation-related protein high mobility group box-1 (HMGB-1) in blood compared to children with fever alone (Lahat et al. 1997; Tomoum et al. 2007; Choi et al. 2011). Experimental data supports the fundamental role of proinflammatory cytokines in febrile seizures: IL-1*β* signaling appears to be integrally involved in the generation of FS in mouse models, where it may act both as a pyrogen and direct proconvulsant, capable of inducing seizures at high doses in the absence of hyperthermia (Dube et al. 2005). The causal link between the rise in cytokines and an increase in seizure susceptibility has been further demonstrated by the finding that a subconvulsive dose of KA followed by intracerebroventricular administration of IL-1*β* increased the proportion of animals experiencing convulsive seizures (Heida and Pittman 2005). In the present study, IL-1*β*, IL-6, and TNF-*α* were elevated in blood plasma. These proinflammatory cytokines are known to be induced by LPS (Rossol et al. 2011) and to act as endogenous pyrogens (Dinarello 1999). They are also released by microglia (Hanisch 2002; Smith et al. 2012). Although IL-1*β* has been shown to be upregulated in the brain of rat pups 24 h after prolonged (64 min) hyperthermia only (Dube et al. 2010), upregulation of proinflammatory cytokines in the blood has not previously been demonstrated in an experimental model after 30 min of hyperthermia. We were able to detect increased levels of these cytokines in the blood of LPS + HT but not HT-only animals following <30 min seizures. This raises the possibility that blood cytokine levels might be appropriate to investigate as candidate predictive biomarkers for at-risk infants for the future development of temporal lobe epilepsy following early life febrile status epilepticus.

Microglia, the only resident antigen-presenting cells in the brain, were significantly activated following LPS + HT compared to HT-only. Microglia have been shown to be activated in various experimental models of seizures (Vezzani et al. 1999; Drage et al. 2002; Avignone et al. 2008; Fabene et al. 2010; Yang et al. 2010) and in chronic drug-resistant epilepsy due to diverse etiology (Beach et al. 1995; Choi et al. 2009). Activated microglia initiate the proinflammatory cytokine cascade and enhance neuronal excitability, thus increasing seizure susceptibility (Somera-Molina et al. 2007, 2009; Kazl et al. 2009; Abraham et al. 2012). Cytokine production induced by microglia may also contribute to pathological changes associated with prolonged seizures, such as reactive gliosis, mossy fiber sprouting, neuronal death, and hippocampal sclerosis (Beach et al. 1995; Jankowsky and Patterson 2001).

In conclusion, systemic injection of LPS simulates fever and primes the brain to more rapidly respond to hyperthermia and to produce more severe seizures. Establishment of this clinically relevant model of infection-associated FS in the rat and mouse may help identify biomarkers for at-risk individuals for the subsequent development of temporal lobe epilepsy and will allow for the use of transgenic technologies to assist in the investigation of signaling pathways for improved targeted therapies after prolonged FS.
